# Supplementary material for: Benevolent Characteristics Promote Cooperative Behaviour among Humans
Source: PLoS One. 2014 Aug 20;9(8):e102881. doi: 10.1371/journal.pone.0102881 (PMC4139200; doi:10.1371/journal.pone.0102881)
Supplement: File S1 — Experimental setup. (PDF) [file pone.0102881.s001.pdf]

Appendix: Experimental setup

We recruited US subjects using the online labor market Amazon Mechanical Turk (AMT) [1–3]. As in classical lab experiments, AMT workers receive a baseline payment and can earn an additional bonus depending on how they perform in the game. AMT experiments are easy to implement and cheap to realise, since AMT workers are paid a substantially smaller amount of money than people participating in physical lab experiments. Nevertheless, it has been shown that data gathered using AMT agree both qualitatively and quantitatively with data collected in physical labs [2–5].

Our experiment was conducted in two sessions. The first session corresponds to Study 1 and Study 2 in the main text, while the second session corresponds to Study 3.

First Session. Subjects were paid a \$0.20 show-up fee for participating and then randomly assigned to one of eight treatments.

T1. Subjects first play BT(\$0.10, \$0.10) as Player 1 and then play PD(\$0.25, \$0.10).
T2. Subjects first play BT(\$0.10, \$1) as Player 1 and then play PD(\$0.25, \$0.10).
T3. Subjects first play BT(\$0.10, \$0.10) as Player 2 and then play PD(\$0.25, \$0.10).
T4. Subjects first play BT(\$0.10, \$1) as Player 2 and then play PD(\$0.25, \$0.10).

Each treatment consists of two economic games. Subjects were informed that computation and payment of the bonuses would be made at the end of the experiment. So, in each treatment, participants played the second game without knowing the outcome of the first. The reason for doing this is that we want to minimise spill over effects due to the fact that subjects in T1-T4 may behave more or less cooperatively in the second game depending on whether or not they have been recipient of a benevolent act in the first game.

A major issue with AMT is that workers try to play multiple times in order to get a larger bonus and/or play randomly in order to complete the task as soon as they can. To control for these issues is very easy using the Survey builder Qualtrics. At the very beginning of the task we asked for the Turk ID and we automatically excluded from the task people who had already completed it. To address the second issue, we asked comprehension questions for each of the economic games and we automatically excluded from the task workers who fail to correctly answer them. We stress the fact that we decided not to ask technical questions, such as computing the expected payoff of a strategy profile, and preferred to ask qualitative questions in order to make clear the dualities between making a benevolent action or not (in the BT game) or between maximising the total welfare versus maximising one's own payoff (in the PD).

385 US subjects, nearly evenly distributed among the eight treatments, answered correctly all comprehension questions.

Here we report the full instructions for Treatment 1. The instructions of the other treatments were absolutely identical, a part from obvious changes in the parameters. The first two screens do not contain any information about the game and serve us only as control to avoid multiple plays from the same subject and lazy participants who can increase randomness on our data.

Screen 1. In the first screen, participants were welcomed to the game and asked to type their worker ID. This allows us to automatically exclude workers who have already completed the task.

Screen 2. In the second screen, we asked the participant to transcribe a relatively long neutral piece of text. This allows us as to tell computers and humans apart (CAPTCHA) and, at the same time, to exclude lazy workers and minimise randomness in our data. We used a meaningless neutral text in order to avoid framing effects.

In the third screen, people entered the real game. Here is the exact instructions we used.

Screen 3. Welcome to this HIT. This HIT has two parts. We will tell you about the second part after you have completed the first one. For your participation in this HIT, you receive $0.20. You also can earn up to $1.20 as a bonus depending on the group to which you are assigned. Full details will be given in the following pages. The size of your bonus depends on the decisions you will make in the tasks that follow but also on the decisions of another anonymous MTurk participant, with whom you are paired. Your bonus will be paid within 10 days of the completion of the HIT. You can opt out at any time although if you choose to exercise this option before completing the HIT then you will not receive any payment or bonus. You will be told about the outcome of all parts of the HIT at the same time that your bonus is paid. If you answer any comprehension question incorrectly the survey will end and unfortunately you will not receive any payment or bonus. With this in mind, do you wish to continue?

Here participants could either continue or end the survey, clicking on the corresponding button. Participants who decided to continue were directed to the next screen.

Screen 4. This is the first part of the HIT. You have been paired with another anonymous participant, and you now have a decision to make. Your decision will NOT affect how much money you earn, but it may affect the other participant's income. Along with your payment for participating in this HIT you are given 10 additional cents as a bonus, which you will keep regardless of what follows (congratulations!), the other participant has been given nothing. You have to choose an amount between 0c and 10c corresponding to the maximum amount of money the other participant can make. The other participant will try to guess your choice. If the other participant's guess is smaller than or equal to the number you have chosen, then they will earn an amount of money equal to that guess. Otherwise they will get nothing. The other person is REAL and will really make a decision. Now we will ask you some questions to make sure you understand the task. You MUST answer all questions correctly to receive any payment or bonus. If you answer incorrectly the survey will terminate and you will not receive a redemption code. There is no incorrect answer when you are asked to make your actual decision.

(1) Which of the following choices made by YOU favours the OTHER PARTICIPANT the most?

(2) Which of the following choices made by YOU minimises the amount the OTHER PARTICIPANT can win?

In both questions, participants could select all possible integers between 0 and 10, in T1, or all possible multiples of ten between 0 and 100, in T2. Questions contained a

Skip Logic, that is a program that automatically ends the survey if the answer is not correct.

Screen 5. Now it's time to really make your decision. Choose an amount from the following list.

The list of possible choices in T1 contained all integers between 0 and 10, while in T2 contained all multiples of 10 between 0 and 100.

Screen 6. This is the second part of the HIT. You are paired with another anonymous participant. The amount of money you earn depends on your choice AND on the choice of the other participant. You and the other participant are both given 10c and you both have the same decision to make. You can either keep the 10 cents or give it all to the other participant who will also receive an extra 5c from us for a total of 15c. So if you both keep the money, you both earn 10c. If you both give all of your money, you will both earn 15c. If you keep all of your money and the other participant gives all of their money, you will earn 25c. If you give all of your money and the other participant keeps all of theirs, then you will earn nothing. The other person is REAL and will really make a decision. Now we will ask you some questions to make sure you understand the task. You MUST answer all questions CORRECTLY to receive any payment or bonus. If you answer incorrectly the survey will terminate and you will not receive a redemption code. There is no incorrect answer when you are asked to make your actual decision.

(1) If the other participant chooses to 'Keep', which choice made by YOU maximises YOUR bonus?

(2) If the other participant chooses to 'Give', which choice by YOU maximises the OTHER PARTICIPANT's bonus?

(3) If you choose to 'Give', which choice by the OTHER PARTICIPANT maximises the OTHER PARTICIPANT's bonus?

(4) If you choose to 'Keep', which choice by the OTHER PARTICIPANT maximises YOUR bonus?

In all questions, participants could select either 'keep' or 'give'. All questions contained a Skip Logic, that is a program that automatically ends the survey if the answer is not correct.

Screen 7. Now it's time to make your decision. What is your choice?

Here participants were asked to either 'keep' or 'give'. No intermediate choices as in [6] were allowed. Following this screen, we asked demographic questions. A final screen, providing a completion code to claim for their payment, concluded the survey.

Second Session. Subjects were paid a $0.20 show-up fee for participating and then randomly assigned to one of three treatments.

T5. After entering the game, participants see a screen where we define benevolence as giving a benefit to someone else at negligible cost to themselves. Subjects are then asked to write a paragraph describing a time when acting benevolently led them in the right direction and resulted in a positive outcome for them. Alternatively, they could write a paragraph describing a time when acting malevolently led them in the wrong

direction and resulted in a negative outcome for them. After this, they are asked to play PD(0.25, 0.10).

T6. This treatment is very similar to T5, with the only difference that subjects are primed towards malevolence. We first define malevolence as an unkind act towards someone else with no immediate benefit for themselves and then we ask participants to write a paragraph describing a time when acting benevolently led them in the wrong direction and resulted in a negative outcome for them. Alternatively, they could write a paragraph describing a time when acting malevolently led them in the right direction and resulted in a positive outcome for them.

T7. This is a baseline treatment, where participants, after entering the game, are immediately asked to play PD(0.25, 0.10), using literally the same instructions as in T5 and T6, in order to avoid framing effects.

In order not to destroy the priming effect we decided not to ask for comprehension questions before the Prisoner's dilemma in T5 and T6. Further, we asked no comprehension questions in T7 so as not to bias any baseline measurement. To control for good quality results we asked the players to describe the reason of their choice. This, together with the descriptions of benevolent or malevolent actions allowed us to manually exclude from the analysis those subjects who did not take the game seriously or showed a clear misunderstanding of the rules of the game. We excluded 11 subjects from the analysis. 300 Subjects, nearly evenly distributed among the three treatments, passed our manual screening. Now we report the exact instructions we used in T5. Those of the other two treatments were essentially the same, a part from obvious changes. Since the first three screens were basically the same as the first three screens in the first session, we start from Screen 4.

Screen 4. Benevolence is defined as giving a benefit to someone else at negligible cost to yourself. Please write a paragraph describing a time when acting benevolently led you in the right direction and resulted in a positive outcome for you. Alternatively, write a paragraph describing a time when acting malevolently led you in the wrong direction and resulted in a negative outcome for you. Of course, anything you write will be treated in the strictest confidence.

Screen 5. Now you are asked to make a decision. You are paired with another anonymous worker. You can earn a bonus depending on your and the other participant's decision. You and the other participant are both given 10c and you both have the same decision to make. You can either keep the 10c or give it to the other participant. In this case we will multiply that amount by 2 and the other participant will earn 20c. So, if you both keep the money, you will earn 10c each; if you both give all of your money, you will earn 20c each; if you keep all of your money and the other participant gives all of their money, you will earn 30c; if you give all of your money and the other participant keeps all of theirs, then you will earn nothing. The other person is REAL and will really make a decision.

Screen 6. What is your choice?

Here participants could select to either 'Give' or 'Keep'. No intermediate choices were allowed. After making their choice, subjects entered the demographic questionnaire. One of the questions was to describe the reason of their choice.

References

[1] Paolacci G, Chandler J, and Ipeirotis PG (2010) Running experiments on amazon mechanical turk. Judgm Decis Mak 5, 411-419.

[2] Horton JJ, Rand DG, and Zeckhauser RJ (2011) The online laboratory: Conducting experiments in a real labor market. Exper Econ 14, 399-425.

[3] Rand DG (2012) The promise of mechanical turk: How online labor markets can help theorists run behavioral experiments. J Theor Biol 299, 172-179.

[4] Suri S and Watts DJ (2011) Cooperation and contagion in web-based, networked public goods experiments. PLoS ONE 6(3):e16836..

[5] Rand DG, Arbesman S, and Christakis NA (2011). Dynamic social networks promote cooperation in ex- periments with humans. PNAS 108 (48) 18, 9193-1919.

[6] Capraro V, Jordan JJ, and Rand DG (2014) Cooperation increases with the benefit-to-cost ratio in one-shot prisoners dilemma experiments. Available at SSRN: http://ssrn.com/abstract=2429862.
